# Pattern and causes of missed appointments in a Nigerian Psychiatric Hospital: A cross-sectional study

**DOI:** 10.1097/MD.0000000000038564

**Published:** 2024-06-14

**Authors:** Bassey Eyo Edet, Emmanuel Aniekan Essien, Emmanuel Omamurhomu Olose, Chidi John Okafor, Molly Unoh Ogbodum, Faithful Miebaka Daniel

**Affiliations:** a Department of Clinical Services, Federal Neuropsychiatric Hospital, Calabar, Nigeria; b Department of Clinical Services, Federal Neuropsychiatric Hospital, Calabar, Nigeria; c Department of Psychiatry, University of Calabar, Calabar, Nigeria; d Department of Public Health, University of Calabar, Calabar, Nigeria; e Community and Clinical Research Division, First On-Call Initiative, Kharkiv Oblast, Ukraine.

**Keywords:** appointment and schedules, psychiatry, treatment adherence and compliance

## Abstract

Psychiatric patients exhibit a higher rate of missed appointments compared to other medical specialities, leading to provider frustration, increased relapse, and suboptimal outcomes. This study investigates the patterns and correlates of missed appointments among outpatients at the Federal Neuropsychiatric Hospital in Calabar, Nigeria. A cross-sectional study involving 403 consecutive outpatient clinic attendees was conducted. The study questionnaire inquired about sociodemographic characteristics and hospital utilization. The Oslo Social Support Scale, the Internalized Stigma of Mental Illness Scale, the Perceived Devaluation and Discrimination Scale, and the Treatment Perception Questionnaire were administered. The mean participant age was 36.19 years (SD = 11.25), with females constituting 52.6%. Missed appointments occurred in 16.6%. The primary reasons for missed appointments included financial difficulties, forgetfulness, and distance to the hospital. Factors significantly associated with missed appointments were marital status (married), having children, believing appointments were too frequent, medication nonadherence, and concerns about medication cost (*P* < .05). Additionally, individuals who received unorthodox or delayed traditional care during their first mental health episode were more likely to miss appointments (*P* < .05). Missed appointments are prevalent among psychiatric patients, often attributed to financial challenges, forgetfulness, and geographical barriers to the hospital. Some of these factors are modifiable, suggesting targeted interventions in adherence improvement programs are needed.

## 1. Introduction

Mental, neurological, and substance use (MNS) disorders contribute about 14% to the global disease burden in high and low-income countries.^[1]^ Despite being globally recognized as a crucial public health concern, it is less prioritized in sub-Saharan Africa, which bears more than 70% of the global mental health burden.^[1]^ Treatment coverage remains notably insufficient in the region, with a gap often exceeding 90%.^[2]^

In Nigeria, <10% access needed care despite the availability of effective and affordable treatments.^[1,3]^ Human resource limitations further contribute to the deficit in treatment coverage. Compared to a global average of 9.0, the African region has 1.4 mental health workers per 100,000 people.^[4]^ Other problems persist, such as inadequate government commitment, a lack of enabling policy or legislation, and poor financing. In addition, specialist mental health care concentrates in urban areas, limiting accessibility for most of the population.^[1]^ Travelling long distances for treatment discourages health-seeking and treatment adherence.^[5]^

Mental disorders are typically chronic conditions requiring long-term care, and patient compliance is essential for optimal clinical outcomes.^[5]^ Unfortunately, psychiatric patients exhibit a 2-fold higher rate of missed appointments compared to other medical specialities.^[5]^ Patients who miss appointments benefit suboptimally from clinical supervision and are less likely to make informed decisions about their care.^[5]^ Furthermore, poor attendance translates to higher healthcare expenses due to illness exacerbation, frequent relapses, re-hospitalization, greater risk of assault, risky behaviors, self-harm, and poorer outcomes.^[6,7]^ Missed appointments can also negatively impact the quality of care, hindering patient–provider communication, fostering provider frustration, and weakening the therapeutic relationship.^[5,6,8]^ Many factors hinder hospital attendance in psychiatric patients, such as financial costs, lack of transportation, inconvenient appointments, distance, and stigma, among others.^[5]^ Elucidating these factors in research is vital for policy development and service reorganization for an improved reach.

Addressing the limited access to mental healthcare in Nigeria requires innovative solutions. Integrating psychiatric care into Primary Healthcare Centers (PHCs) holds the greatest potential for reaching most of the Nigerian population, particularly those residing in rural and suburban areas who rely solely on this tier of care.^[9]^ The 2013 Policy on Mental Health Service Delivery in Nigeria not only advocates for this integration but also offers guidelines for successful implementation across all 3 tiers of healthcare delivery.^[9]^ However, only 3 of Nigeria’s 36 states (Osun, Ogun, and Lagos) have integrated mental health services within their PHCs.^[9]^

This study was conducted as baseline research for developing community mental health services within Cross River State in Nigeria’s South–South region. It aimed to investigate the pattern of missed appointments and establish the common reasons and correlates of missed appointments among patients attending the outpatient clinic. The anticipated outcomes of this research were to uncover barriers to accessing services in the local psychiatric hospital, potentially justifying the establishment’s decision to integrate mental health care into Primary Health Centers (PHCs). This baseline data would also be utilized to advocate for the government and key stakeholders to support and fund the project. Additionally, the data generated by this research could inform local hospital policies and interventions for improving hospital attendance. Furthermore, our findings might apply to other psychiatric hospitals in Nigeria, as they serve a comparable population within a similar socioeconomic and cultural terrain. Our results could offer them valuable insights for similar interventions to integrate mental health services into PHCs or improve treatment adherence.

## 2. Materials and methods

### 2.1. Study design

This was a cross-sectional study among outpatients of the Federal Neuropsychiatric Hospital, Calabar. The study strictly adhered to the guidelines for strengthening the reporting of observational studies.^[10]^

### 2.2. Setting

Cross River is one of Nigeria’s 36 states, covering a landmass of 20,156 km² and divided into 3 senatorial districts: South (comprised of Calabar Municipality, Calabar South, Akamkpa, Akpabuyo, Bakassi, Biase, and Odukpani Local Government Areas), Central (comprised of Abi, Yakurr, Obubra, Ikom, Etung, and Boki Local Government Areas), and the North (Bekwarra, Obanliku, Obudu, Ogoja, Yala Local Government Areas).

The Federal Neuropsychiatric Hospital is located in Calabar South Local Government Area (LGA). It currently has 200 beds and provides mental health services to Cross River State, which has a population of about 4 million. The hospital’s reach extends beyond the state, catering to patients from the catchment area, including Benue, Akwa Ibom, Abia, Ebonyi, and even Cameroon. Some have to travel long distances to reach the hospital. For example, patients from Obudu LGA (located within the state) travel approximately 428 km in a 6-hour journey to access hospital services. Data were collected in the outpatient clinic of the hospital in November 2021.

### 2.3. Study participants

All patients attending the clinic who were over 18 years of age, registered as patients for at least 1 year, and stable enough to fill study questionnaires were considered eligible for recruitment.

### 2.4. Sample size calculation

The Cochran formula was employed using a known proportion^[11]^ to estimate the sample size at 95% confidence interval (CI) and 5% precision level.^[12]^ This resulted in a sample size of 374. To account for potential nonresponse, we increased the sample size by 10%, bringing the final sample size to 411.

### 2.5. Study variables

Data were collected using self-report questionnaires in the following sections.

1. *Section A:* This assessed sociodemographic variables, including age, gender, marital status, and indicators of socioeconomic status, including education, employment, and income.
2. *Section B:* Variables on hospital service utilization were assessed in this section. It included a list of possible reasons for missed appointments, such as the regularity of hospital appointments, perception of appointment frequency, transportation cost, hospital trip duration, and drug cost. Researchers generated the list based on previous research and their experience with psychiatric patients over several years of practice. Respondents were also allowed to fill in other unlisted reasons as applicable.

A question in this section (How often do you miss your hospital appointment?) assessed the rate of missed appointments. Possible responses were on a 5-point Likert scale ranging from *Very Frequently, Frequently, Occasionally, Rarely, Very Rarely, Never.* In this study, “missed appointments” were operationally defined as those who missed appointments *very frequently, frequently or occasionally*.

3. *Section C:* This section included the following instruments.
  a. *MacArthur Scale of Subjective Social Status:* This visual analog scale is a 10-rung ladder that measures socioeconomic status.^[13]^ Higher rungs indicate affluence, while lower rungs reflect a low socioeconomic status. The scale does not have standardized cutoffs and was treated as a continuous variable in the analysis. It is a valid measure of social class with good psychometric properties.^[14]^
  b. *Oslo Social Support Scale (OSS):* This scale assessed respondents’ social support. It is a 3-item measure that inquires about the concern shown by others, the number of people that can be reached in times of crisis and the ease of getting help from neighbors. Low scores suggest a low level of social support. The OSS is a valuable tool due to its brevity and well-established psychometric properties.^[15]^
  c. *Internalized Stigma of Mental Illness (ISMI-10) scale:* This is a 10-item measure of self-stigma, i.e. the extent to which patients stigmatize themselves.^[16]^ It has a 5-point Likert scale ranging from strongly disagree to strongly agree, and higher scores indicate a greater level of internalized stigma. ISMI has been used in many countries and is reliable and valid.^[16]^
  d. *Perceived Devaluation and Discrimination Scale (PDD):* This instrument was used to measure the extent to which respondents believe others will discriminate against people with a mental disorder.^[17]^ It has 12 items using a 6-point Likert scale requiring responses ranging from strongly agree to strongly disagree. A high score indicates a higher perception of discrimination evaluation, and the scale has been shown to have acceptable reliability and validity.^[17,18]^
  e. *Treatment Perception Questionnaire (TPQ):* This self-report tool assessed patient satisfaction with services. It consists of ten items rated on a Likert scale with options ranging from strongly agree to strongly disagree.^[19]^ It was chosen because of its brevity and has been shown to have good psychometric properties. Higher scores indicate greater satisfaction with services.^[19]^

### 2.6. Study procedure

On clinic days, respondents were approached during wait time for participation. The aims and objectives were explained, and consenting patients were consecutively recruited. This procedure, which included administering study questionnaires, was done by trained research assistants. Although the consecutive sampling technique was employed, bias was reduced by a strict and uniform application of inclusion and exclusion criteria and sampling over various clinic cycles, capturing a wide demographic range and reducing temporal bias. Furthermore, we employed research assistants for questionnaire administration, with researchers remaining blind to this process, adding a layer of objectivity.

### 2.7. Ethical consideration

The research received approval from the Ethical Research Committee of the Federal Neuropsychiatric Hospital in Calabar, Nigeria. All participants provided informed consent before their inclusion. Furthermore, this study strictly followed the Helsinki Declaration’s principles regarding human subject research.

### 2.8. Data analysis

IBM SPSS Statistics for Windows, version 22.0 (IBM Corp; Armonk, NY, USA) was used for data analysis. Sociodemographic variables were expressed as frequencies and proportions. Pearson chi-square test and Student *t* test were done for bivariate analysis. The alpha level was set at 0.05.

## 3. Results

A total of 411 questionnaires were distributed, with 8 excluded due to incomplete data, resulting in a final sample size of 403 participants. As seen in Table 1, most respondents were over 45 years of age, with a relatively equal distribution of males and females. About a third were married, and almost half had attained tertiary education. Over 50% were employed, but the overwhelming majority earned <50,000 Naira monthly. Notably, most respondents lived in the city of Calabar.

**Table 1:** Sociodemographic and missed appointment variables.

Variable	Frequency (n = 403)	Percentage (100%)
Age		
18–25	70	17.4
26–35	133	33.0
36–45	136	33.7
46–55	38	9.4
Above 55	26	6.5
Mean 36.19 [SD = 11.25]		
Gender		
Male	191	47.4
Female	212	52.6
Marital status		
Married	132	32.8
Sep/Div	76	18.9
Single	195	48.4
Education		
Primary	55	13.6
Secondary	162	40.2
Tertiary	186	46.2
Employment		
Employed	225	55.8
Unemployed	178	44.2
Monthly income		
<50,000 naira	319	79.2
50,000 to 100,000 naira	71	17.6
Above 100,000	13	3.2
Self-rated affluence		
Low	133	33.0
Average	155	38.5
High	115	28.5
State of residence		
Calabar	237	58.8
Outside Calabar but within the State	122	30.3
Outside the State	38	9.4
Missed appointments		
Very Frequently	2	0.5
Frequently	14	3.5
Occasionally	51	12.7
Rarely	43	10.7
Very rarely	53	13.2
Never	240	59.6

SD = standard deviation.

After categorization based on operational definition, 67 (16.6%) had missed appointments. Reasons why patients miss their appointments, in order of frequency, were as follows: *Financial problems* in 79 cases (19.6%); *forgetting* in 44 (10.9%), *hospital distance* in 33 (8.2%), *school or work demands* in 19 (4.7%), *poor quality of service* in 11 (2.7%), *visits being too frequent* in 10 (2.5%), *attendance being an embarrassment to family* in 9 (2.2%), *belief that their illness is spiritually caused* in 9 (2.2%), *to avoid being identified as mentally ill* in 8 (2.0%), *due to other competing engagements* in 8 (2.0%), *feeling ashamed to attend* in 7 (1.7%), *time wastage by visits* in 7 (1.7%), *low-income family support* in 7 (1.7%), *treating self by other means* in 7 (17%), and *missing appointments after remission* in 4 cases (1.0%).

Tables 2–4 show the results of bivariate analyses to determine the correlates of missed appointments. Being married, having children, believing appointments were too often, perceiving medication as expensive, and missing medication frequently were significantly associated with missing appointments (*P* < .05). Interestingly, participants who initially received unorthodox care or experienced delays in receiving traditional care during their first mental health episode were also more likely to miss appointments (*P* < .05). Other variables such as employment status, income, residence, being accompanied by a relative, hospital trip duration, transportation cost, social support, self-stigma, perceived stigma and service satisfaction were not significantly associated with missed appointments (*P* < .05).

**Table 2:** Sociodemographic correlates of missed appointment.

Variable	Missed appointment		cOR (95% CI)	Statistic
	No	Yes		
Age				
35 or less	175 (86.2)	28 (13.8)	1.15 (.89–2.57)	*X*^2^ = 2.36
Above 35	161 (80.5)	39 (19.5)	Ref	*P* = .12
Gender				
Male	166 (86.9)	25 (13.1)	1.64 (.95–2.81)	*X*^2^ = 3.27
Female	170 (80.2)	42 (19.8)	Ref	*P* = .07
Marital status				
Married	102 (77.3)	30 (22.7)	1.86 (1.09–3.17)	*X*^2^ = 5.27
Unmarried	234 (86.3)	37 (13.7)	Ref	*P* = **.02**
Education				
Primary	43 (78.2)	12 (21.8)	1.34 (.63–2.82)	*X*^2^ = 1.80
Secondary	139 (85.8)	23 (14.2)	.79 (.44–1.42)	*P* = .40
Tertiary	154 (82.8)	32 (17.2)	Ref	
Employment				
Employed	190 (84.4)	35 (15.6)	Ref	*X*^2^ = 0.42
Unemployed	146 (82.0)	32 (18.0)	1.19 (.70–.20)	*P* = .51
Monthly income				
<50,000 naira	263 (82.4)	56 (17.6)	Ref	
50,000–100,000 naira	62 (87.3)	9 (12.7)	.68 (.32–1.45)	*X*^2^ = 1.01
Above 100,000	11 (84.6)	2 (15.4)	.85 (.18–3.9)	*P* = .60
Residence				
Calabar city	196 (82.7)	41 (17.3)	Ref	*X*^2^ = 1.70
Outside Calabar city	134 (84.3)	25 (15.7)	.68 (.89–.51)	*P* = .68
Have children				
No	161 (88.5)	21 (11.5)	Ref	*X*^2^ = 6.19
Yes	175 (79.2)	46 (20.8)	2.01 (1.15–3.52)	*P* = **.01**

Bold values are statistically significant (*P* < 0.05).

CI = confidence interval, OR = odds ratio.

**Table 3:** Other correlates of missed appointment.

VARIABLE	Missed appointment	cOR (95% CI)	Statistic	X^2^ = 0.46 *P* = .56
	No	Yes		
Appointment regularity				
Monthly or less	230 (84.2)	43 (15.8)	Ref	*X*^2^ = 0.46
2 monthly or more	106 (81.5)	24 (18.5)	1.21 (.69–2.09)	*P* = .56
Believes visit is too frequent				
Yes	55 (66.3)	28 (33.7)	3.66 (2.08–6.45)	*X*^2^ = 22.07
No	281 (87.8)	39 (12.2)	Ref	*P* = **.000**
Accompanied for visit				
Sometimes to always	252 (81.8)	56 (18.2)	1.67 (.85–3.39)	*X*^2^ = 2.22
Rarely to never	84 (88.4)	11 (11.6)	Ref	*P* = .15
Who pays transportation				
Self	172 (81.5)	39 (18.5)	1.32 (.78–2.25)	*X*^2^ = 1.10
Other	164 (85.4)	28 (14.6)	Ref	*P* = .29
Miss school or work				
Yes	160 (81.2)	37 (18.8)	1.35 (.80–8.29)	*X*^2^ = 1.23
No	176 (85.4)	30 (14.6)		*P* = .25
Frequency of missing drugs				
Rarely–never	316 (90.0)	35 (10.0)	Ref	*X*^2^ = 86.88
Occasionally–often	20 (38.5)	32 (61.5)	14.44 (7.45–27.91)	*P* = **.000**
Drug cost				
Affordable	115 (87.6)	22 (12.4)	Ref	*X*^2^ = 4.00
Expensive	181 (80.1)	45 (19.9)	1.75 (1.00–3.04)	*P* = **.04**
Time to access care at onset				
Within 1 mo	190 (88.4)	25 (11.6)	2.72 (1.53–4.85)	*X*^2^ = 12.19
Over a month	92 (73.6)	33 (26.4)	Ref	*P* = **.000**
Place of first care at onset				
Orthodox	285 (85.1)	50 (14.9)	Ref	*X*^2^ = 4.13
Unorthodox	51 (75.0)	17 (25.0)	1.90 (1.01–3.55)	*P* = **.04**
Willing to visit get reminders				
Yes	97 (78.2)	27 (21.8)	Ref	*X*^2^ = 3.42
No	239 (85.7)	40 (14.3)	1.66 (.96–2.86)	*P* = .06

Bold values are statistically significant (*P* < 0.05).

CI = confidence interval, OR = odds ratio.

**Table 4:** Other correlates of missed appointment continued.

Variable	Missed appointment		Statistic	MD (95% CI)
	No Mean (SD)	Yes Mean (SD)		
Time to Hospital	85.84 (138.14)	90.14 (182.33)	*T* = −.22	−4.30 (−42.79 to 34.18)
			*P* = .82	
Transport	937.81 (2200.12)	792.31 (1183.78)	*T* = .51	145.50 (−406.77 to –697.78)
			*P* = .60	
Affluence	4.31 (1.52)	4.37 (1.79)	*T* = −.27	−.07 (−.64 to .48)
			*P* = .78	
Treatment duration (in yrs)	5.77 (6.28)	6.73 (6.54)	*T* = −1.13	−.96 (−2.62 to .70)
			*P* = .25	
Drug expenditure per visit	3900.17 (2885.84)	4192.06 (2892.68)	*T* = −.73	−291.89 (−1082.06 to 498.27)
			*P* = .46	
Transport when accompanied	1883.80 (4334.41)	1710.98 (2279.35)	*T* = .24	172.82 (−743.82 to 1089.46)
			*P* = .80	
OSS	10.39 (2.33)	10.50 (2.05)	*T* = −0.36	−.11 (−.71 to .49)
			*P* = .71	
PDD	31.7 (4.84)	31.2 (4.64)	*T* = .85	.55 (−.71 to 1.81)
			*P* = .39	
ISMI	27.99 (4.64)	27.13 (4.44)	*T* = 1.39	.85 (−.35 to 2.06)
			*P* = .16	
TPQ	28.44 (5.38)	28.61 (5.20)	*T* = −.22	−.16 (−1.57 to 1.24)
			*P* = .82	

CI = confidence interval, MD = mean difference, OSS = Oslo Social Support Scale, PDD = perceived discrimination devaluation scale, TPQ = Treatment Perception Questionnaire.

## 4. Discussion

This study investigated the patterns and factors associated with missed appointments among outpatients attending a psychiatric hospital in Nigeria. Based on our operational definition, we found that less than a fifth (16.6%) of participants missed their appointments. The primary reasons for missed appointments included forgetting, financial difficulty, and hospital distance. Variables associated with missed appointments were marital status, having children, perceptions concerning the cost and regularity of treatment, receiving unorthodox care or delayed care in the first episode of illness, and medication adherence.

The measurement of missed appointments varies widely across studies, which limits direct comparison.^[20]^ However, our prevalence falls within the reported range (8.1–46.2%) in previous research.^[11,21]^ Despite the variation in estimates, missed appointments are generally higher among individuals with mental illness than nonpsychiatric populations and constitute a public health challenge.^[5]^

The reasons identified for missing appointments were broadly similar to previous research.^[11,22–24]^ Forgetting appointments, although most common in other studies, ranked second in our findings.^[11,22]^ Financial difficulty emerged as the top reason for missed appointments in our study, reflecting a challenge common in resource-constrained settings like Nigeria, where out-of-pocket payments are the norm.^[11,22]^ This contrasts with studies conducted in developed nations like the USA and Taiwan, where health insurance likely mitigates financial barriers.^[25,26]^ Additionally, consistent with prior research, hospital distance was another significant factor associated with missed appointments.^[11,23]^

Missed appointments were higher among the married, consistent with findings from an Indian study^[27]^; however, most studies do not report a relationship.^[22]^ Contrarily, some Nigerian studies found that the unmarried were more likely to miss appointments and surmised that the social support from marriage improved clinic attendance.^[6,23]^ While this proposition appears plausible, it is essential to acknowledge that married individuals contend with additional family responsibilities, potentially impeding their ability to attend clinics consistently. Supporting this notion, we found that respondents with children also had poorer attendance. Disparities in how missed appointments are measured across studies could contribute to variations in findings.

We expected an association between missing appointments and socioeconomic status indicators such as education, employment, income, and affluence. This would have supported our finding that financial difficulty was the most common reason for missed appointments. Some previous reports found an association with socioeconomic factors,^[20,23,28]^ but others revealed none.^[22,29,30]^ Interestingly, our study found that patients who believed medications were expensive were more likely to miss appointments. While this suggests a potential role for socioeconomic factors, it might highlight the relative importance of perception rather than the actual cost of treatment. Social inequalities are known to affect healthcare access.^[31]^ Our approach to measuring missing appointments, which was subjective rather than objective, might account for the non-significance.

Contrary to previous reports, other variables that strain resources, such as distance, transportation cost, and hospital trip duration, were unrelated to missing appointments.^[20,23,32,33]^ This could again be attributed to our reliance on self-reported missed appointments. Similarly, the frequency of visits also showed statistical non-significance. However, believing hospital visits were too frequent significantly correlated with missed appointments. This finding underscores the importance of considering patient attitudes toward treatment requirements and their healthcare experience.

Consistent with previous research, our study found a link between medication nonadherence and missed appointments.^[34,35]^ Both are treatment adherence variables, and their correlation is expected. Factors that determine hospital attendance might also affect compliance with medication. Furthermore, patients who miss their medication might be more likely to relapse, increasing the likelihood of missing appointments.

Delayed access to care at illness onset was associated with missed appointments, suggesting that barriers to the first hospital contact might impede subsequent contact as well. In this study, patients who received unorthodox care in their first episode had more missed appointments. Cultural norms and competing traditional models of care are known barriers to accessing mental health services.^[36]^ Beliefs that mental illness has a spiritual cause or doubts about conventional care might lead to a preference for alternative treatments.

While those who missed appointments expressed a greater willingness for reminders compared to regular attendees, this difference was not statistically significant. Even so, this information is clinically valuable, especially as forgetting was one of the most common reasons for missed appointments. Those who forget their appointment dates probably recognize that reminders would be helpful, which might explain their willingness.

Consistent with previous research, social support was not associated with missing appointments, but this was somewhat unexpected.^[37–39]^ Family support reduces relapse and improves clinical outcomes.^[40]^ Our instrument may not have captured the specific aspects of social support crucial for treatment adherence. Similarly, stigma was not linked to missed appointments, contradicting previous research on its influence on healthcare-seeking behavior among those with mental illness.^[41,42]^ This inconsistency may be due to how we measured missed appointments. Finally, patient satisfaction did not correlate with missed appointments, which also differs from some past studies.^[27,37]^ Since the questionnaires were administered within the hospital, patients might have given more socially desirable responses concerning their service satisfaction.

## 5. Limitations

The study location, sample size, sampling technique, and study design are potential sources of bias that may impact the generalizability of the findings. Efforts were made to mitigate this by standardizing the sample size (exceeding the minimum calculation) and training research assistants to minimize recruitment bias. However, other limitations are worth noting. Using a subjective self-report measure of missed appointments could result in recall bias.

Additionally, asking patients about service utilization while they wait for care might introduce social desirability bias. Furthermore, relying on a single question to measure missed appointments might not provide a complete picture. These limitations could explain the lack of statistical association with factors like hospital distance and transportation costs.

It is important to note that a lack of statistical significance does not necessarily imply these variables have no substantive impact on clinical outcomes. Future research could benefit from adopting a more objective measure of missed appointments or using more comprehensive scales to explore reasons for poor clinic attendance.

## 6. Conclusion and recommendations

This study found that missed appointments are prevalent among psychiatric outpatients. Interventions are critically needed to address reasons for missed appointments and ultimately improve clinic attendance. Our findings suggest several strategies to enhance clinic attendance. Decentralizing psychiatric services by integrating them into primary healthcare settings could lessen the impact of distance and transportation costs, making care more accessible. Additionally, implementing a system for sending appointment reminders could help patients who forget appointments.

Furthermore, adopting a more patient-centered approach is crucial. Increased patient involvement in treatment planning, particularly regarding appointment scheduling, might address concerns about appointment frequency and improve overall treatment engagement. Prioritizing patient psychoeducation can also enhance their understanding of the treatment plan and the rationale behind appointment frequency. Finally, improved mental health advocacy and education for the general population can address socio-cultural norms and health behaviors that hinder access to conventional mental healthcare services.

## Author contributions

**Conceptualization:** Bassey Eyo Edet, Emmanuel Aniekan Essien.

**Supervision:** Bassey Eyo Edet, Emmanuel Aniekan Essien.

**Writing – original draft:** Bassey Eyo Edet, Emmanuel Aniekan Essien, Emmanuel Omamurhomu Olose, Chidi John Okafor, Molly Unoh Ogbodum, Faithful Miebaka Daniel.

**Writing – review & editing:** Bassey Eyo Edet, Emmanuel Aniekan Essien, Emmanuel Omamurhomu Olose, Chidi John Okafor, Molly Unoh Ogbodum, Faithful Miebaka Daniel.

**Data curation:** Emmanuel Aniekan Essien.

**Formal analysis:** Emmanuel Aniekan Essien.

**Methodology:** Emmanuel Aniekan Essien, Faithful Miebaka Daniel.

**Resources:** Emmanuel Aniekan Essien.

**Software:** Emmanuel Aniekan Essien, Faithful Miebaka Daniel.

**Validation:** Emmanuel Aniekan Essien, Faithful Miebaka Daniel.

**Visualization:** Faithful Miebaka Daniel.

## References

[1] World Health Organization (WHO). Mental Health Gap Action Program – Scaling up Care for Mental, Neurological, and Substance Use Disorders. Geneva, Switzerland: World Health Organisation; 2008.

[2] Whiteford HA Ferrari AJ Degenhardt L. The global burden of mental, neurological and substance use disorders: an analysis from the Global Burden of Disease Study 2010. PLoS One. 2015;10:e0116820–14. 25658103 10.1371/journal.pone.0116820 PMC4320057

[3] Gureje O Uwakwe R Udofia O. Common psychiatric disorders over a lifetime: age of onset, risk and treatment contact in the Nigerian survey of mental health and wellbeing. Afr J Med Med Sci. 2008;37:207–17. 18982812

[4] Sankoh O Sevalie S Weston M. Mental health in Africa. Lancet Glob Health. 2018;6:e954–5. 30103990 10.1016/S2214-109X(18)30303-6

[5] Mitchell AJ Selmes T. Why don’t patients attend their appointments? Maintaining engagement with psychiatric services. Adv Psychiatr Treat. 2007;13:423–34.

[6] James B Omoaregba J Akhigbe S. Prevalence and correlates of missed first appointments among outpatients at a Psychiatric Hospital in Nigeria. Ann Med Health Sci Res. 2014;4:763. 25328790 10.4103/2141-9248.141550 PMC4199171

[7] Centorrino F Hernán MA Drago-Ferrante G. Factors associated with noncompliance with psychiatric outpatient visits. Psychiatr Serv. 2001;52:378–80. 11239109 10.1176/appi.ps.52.3.378

[8] Kruse GR Rohland BM Wu X. factors associated with missed first appointments at a psychiatric clinic. Psychiatr Serv. 2002;53:1173–6. 12221319 10.1176/appi.ps.53.9.1173

[9] Chu C Roxas N Aguocha CM. Integrating mental health into primary care: evaluation of the Health Action for Psychiatric Problems In Nigeria including Epilepsy and SubstanceS (HAPPINESS) pilot project. BMC Health Serv Res. 2022;22:333. 35279154 10.1186/s12913-022-07703-1 PMC8917687

[10] Vandenbroucke JP von Elm E Altman DG.; STROBE initiative. Strengthening the Reporting of Observational Studies in Epidemiology (STROBE): explanation and elaboration. Ann Intern Med. 2007;147:W163–94. 17938389 10.7326/0003-4819-147-8-200710160-00010-w1

[11] Ramlucken L Sibiya MN. Frequency and reasons for missed appointments of outpatient mental health care users in the uMgungundlovu District. Curationis. 2018;41:1–4. 10.4102/curationis.v41i1.1835 PMC6111624 30198291

[12] Cochran WG. Sampling Techniques. New York: John Wiley & Sons; 1977.

[13] Adler N Stewart J. The MacArthur Scale of Subjective Social Status. San Francisco: MacArthur Research Network on SES & Health; 2007.

[14] Chen B Covinsky KE Cenzer IS. Subjective social status and functional decline in older adults. J Gen Intern Med. 2012;27:693–9. 22215272 10.1007/s11606-011-1963-7 PMC3358399

[15] Abiola T Udofia O Zakari M. Psychometric properties of the 3-item Oslo social support scale among clinical students of Bayero University Kano, Nigeria. Malaysian J Psychiatry. 2013;22:32–41.

[16] Boyd JE Otilingam PG DeForge BR. Brief version of the Internalized Stigma of Mental Illness (ISMI) scale: psychometric properties and relationship to depression, self esteem, recovery orientation, empowerment, and perceived devaluation and discrimination. Psychiatr Rehabil J. 2014;37:17–23. 24660946 10.1037/prj0000035

[17] Link BG. Understanding labeling effects in the area of mental disorders: An assessment of the effects of expectations of rejection. Am Sociol Rev. 1987;52:96–112.

[18] Barke A Nyarko S Klecha D. The stigma of mental illness in Southern Ghana: attitudes of the urban population and patients’ views. Soc Psychiatry Psychiatr Epidemiol. 2011;46:1191–202. 20872212 10.1007/s00127-010-0290-3 PMC3192946

[19] Marsden J Stewart D Gossop M. Assessing client satisfaction with treatment for substance use problems and the development of the Treatment Perceptions Questionnaire (TPQ). Addict Res. 2000;8:455–70.

[20] Daniels K Loganathan M Wilson R. Appointment attendance in patients with schizophrenia. Clin Pract. 2014;11:467–82.

[21] Eytan A Gex-Fabry M Ferrero F. Missed appointments at outpatient psychiatric clinics in Geneva: a pilot study. Arch Suisses Neurol Psychiatr. 2004;155:125–8.

[22] Thomas FI Olotu SO Omoaregba JO. Prevalence, factors and reasons associated with missed first appointments among outpatients with schizophrenia at the Federal Neuro-Psychiatric Hospital, Benin City. BJPsych Open. 2018;4:49–54. 29971144 10.1192/bjo.2017.11 PMC6020260

[23] Nwefoh E Aguocha CM Achor J. Missed post-hospitalisation clinic appointment in a psychiatric hospital in Southeast Nigeria. Ment Health Relig Cult. 2018;21:564–77.

[24] Ray S. Identifying barriers to keeping appointments in mental health. Doctor of Nursing Practice Projects; 2021. Available at: https://spark.siue.edu/dnpprojects/171/ [access date February 2, 2022].

[25] Dotter JF Labbate LA. Missed and canceled appointments at a military psychiatry clinic. Mil Med. 1998;163:58–60. 9465575

[26] Chow Y-T Li-Chin Wu MSE Chen C-Y. Who fails appointments at psychiatric clinics in a general hospital in Taiwan? Taiwan J Psychiatry. 2009;23:62–71.

[27] Grover S Mallnaik S Chakrabarti S. Factors associated with dropout from treatment: an exploratory study. Indian J Psychiatry. 2021;63:41–51. 34083819 10.4103/psychiatry.IndianJPsychiatry_87_19 PMC8106432

[28] Lieberman PB Guggenheim FG. Reasons for patient nonattendance during acute partial hospitalisation. Psychiatr Serv. 2016;67:684–7. 26725288 10.1176/appi.ps.201400416

[29] Grunebaum M Luber P Callahan M. Predictors of missed appointments for psychiatric consultations in a primary care clinic. Psychiatr Serv. 1996;47:848–52. 8837157 10.1176/ps.47.8.848

[30] Chantarat W. The reason and impact of missed psychiatric appointment. J Ment Health Thail. 2011;19:148–59.

[31] Baeten R Spasova S Vanhercke B. Inequalities in Access to Healthcare: A Study of National Policies. Brussels: European Commission; 2018.

[32] Charupanit W. Factors related to missed appointment at psychiatric clinic in Songklanagarind Hospital. J Med Assoc Thai. 2009;92:1367–9. 19845246

[33] Singla M Goyal S Sood A. Profile and pattern of follow-ups of psychiatry outpatients at Christian Medical College, Ludhiana. J Ment Health Hum Behav. 2015;20:76.

[34] Alpak G Aksoy I Demir B. Missed appointments and medication non-compliance among consecutive psychiatric patients. J Mood Disord. 2015;5:151.

[35] Miller MJ Ambrose DM. The problem of missed mental healthcare appointments. Clin Schizophr Relat Psychoses. 2019;12:177–84. 27996314

[36] Oguamanam AN Oguamanam GO. Barriers to utilisation of mental health services in Nigeria. Int J Health Soc Inq. 2018;4:58–76.

[37] Adelufosi A Ogunwale A Adeponle A. Pattern of attendance and predictors of default among Nigerian outpatients with schizophrenia. Afr J Psychiatry. 2013;16:283–7. 10.4314/ajpsy.v16i4.38 24051568

[38] Gunzler DD Morris N Dalton JE. Clinic appointment attendance in adults with serious mental illness and diabetes. Am J Health Behav. 2017;41:810–21. 29025509 10.5993/AJHB.41.6.15 PMC6329373

[39] Burton A Walters K Marston L. Is there an association between perceived social support and cardiovascular health behaviours in people with severe mental illnesses? Soc Psychiatry Psychiatr Epidemiol. 2020;55:1659–69. 32424502 10.1007/s00127-020-01879-9 PMC7585561

[40] Simanullang RH. The correlation between family support and relapse in schizophrenia at the psychiatric hospital. Belitung Nurs J. 2018;4:566–71.

[41] Corrigan PW Druss BG Perlick DA. The impact of mental illness stigma on seeking and participating in mental health care. Psychol Sci Public Interest. 2014;15:37–70. 26171956 10.1177/1529100614531398

[42] Rüsch N Angermeyer MC Corrigan PW. Mental illness stigma: concepts, consequences, and initiatives to reduce stigma. Eur Psychiatry. 2005;20:529–39. 16171984 10.1016/j.eurpsy.2005.04.004

